# Stereotactic arrhythmia radioablation: radiobiology, clinical evidence, and emerging applications

**DOI:** 10.3389/fonc.2026.1953364

**Published:** 2026-10-26

**Authors:** Fabiana Gregucci, John Ng, Jim W. Cheung, George Thomas, Christopher F. Liu, Steven M. Markowitz, Fereshteh Talebi, Brendan Roth, Maahi Patel, Ashley Teal, Leland Muller, Xi Kathy Zhou, Ryan Pennell, Silvia C. Formenti

**Affiliations:** 1 Department of Radiation Oncology, Weill Cornell Medicine, NewYork Presbyterian Hospital, New York, NY, United States; 2 Division of Cardiology, Weill Cornell Medicine, NewYork Presbyterian Hospital, New York, NY, United States; 3 Division of Biostatistics, Department of Population Health Sciences, Weill Cornell Medicine, New York, NY, United States

**Keywords:** atrial fibrillation, cardiac radioablation, radiobiology, stereotactic arrhythmia radioablation (STAR), ventricular tachycardia (VT)

## Abstract

STereotactic Arrhythmia Radioablation (STAR) has emerged as a promising non-invasive treatment for patients with refractory cardiac arrhythmias who are unsuitable for or have failed conventional catheter ablation. Initially developed for ventricular tachycardia (VT), STAR has demonstrated encouraging efficacy in reducing arrhythmia burden while avoiding the risks associated with invasive procedures. More recently, limited and early clinical experience has explored the potential application of STAR in paroxysmal atrial fibrillation (AF), including the feasibility of non-invasive pulmonary vein isolation; however, clinical evidence in AF remains anecdotal and investigational. Despite these advances, the biological mechanisms underlying STAR remain incompletely understood. While early hypotheses attributed its therapeutic effects primarily to radiation-induced fibrosis, accumulating evidence suggests that rapid anti-arrhythmic effects are mediated by early electrophysiological reprogramming and conduction remodeling, whereas delayed vascular injury, inflammation, and fibrosis may contribute to durable substrate modification and long-term toxicity. This evolving paradigm has important implications for optimizing radiation dose, fractionation strategies, and minimizing cardiac toxicity. Advances in imaging, electroanatomical mapping, and treatment planning are further improving treatment precision. Ongoing prospective clinical trials and international collaborative initiatives are expected to provide robust evidence regarding efficacy, safety, and long-term outcomes. This review summarizes current knowledge on the radiobiological mechanisms, clinical applications, and future perspectives of STAR, highlighting the key challenges for its integration into contemporary cardiac electrophysiology, as well as describing our current clinical trial investigating the role of dose and fractionation for this technique.

## 1 Introduction

Cardiac arrhythmias, including ventricular tachycardia (VT), remain a major global health burden and despite new treatment advances are associated with reduced quality of life and poor prognosis (1). Therapeutic options include medication, anti-tachycardia pacing or shock by implantable cardiac device (ICD) and catheter ablation (CA) of cardiac arrhythmogenic substrates. CA treatment, which employs radiofrequency (RF) or freezing (cryoablation), is mainstay therapy for drug-refractory VT by generating tissue necrosis and inactivation of the substrate for underlying arrhythmia (2). Limitations of catheter ablation for VT include the inability to create sufficiently deep or transmural lesions across some arrhythmogenic substrates, as well as the inability to safely access certain epicardial substrates. Epicardial access may be particularly challenging in patients with dense pleuropericardial adhesions following previous cardiac surgery, prior chemical or surgical pleurodesis, or previous mediastinal radiotherapy. Similarly, patients with an epicardial arrhythmogenic substrate in whom subxiphoid epicardial access has previously failed or is considered unsafe may have limited conventional treatment options. In these selected clinical scenarios, STereotactic Arrhythmia Radioablation (STAR) may represent a particularly valuable non-invasive alternative because it can target otherwise difficult-to-access myocardial substrates without requiring direct pericardial or myocardial instrumentation (3) hemodynamic. Several advanced interventional strategies have been developed to address these challenges, including bipolar and needle ablation for intramural substrates, as well as pulsed-field ablation and hybrid surgical-catheter approaches for complex endocardial and epicardial substrates (4, 5). These approaches may provide additional treatment options in selected patients. Along these alternative approaches, STAR has emerged as a potential non-invasive option for patients with refractory VT in whom conventional and advanced catheter-based or surgical approaches are unsuccessful, infeasible, or associated with unacceptable procedural risk. Over the past decade, STereotactic Arrhythmia Radioablation (STAR) has emerged as a non-invasive therapeutic option for patients with refractory VT. Early clinical studies have demonstrated substantial reductions in ventricular arrhythmia burden and ICD therapies, accompanied by an acceptable short- to mid-term safety profile (6–10). STAR delivers non-invasive, image-guided, precise, high radiation dose to cardiac target, reducing exposure to adjacent normal tissues. This multidisciplinary treatment involves radiation oncologists, cardiologists, electrophysiologists and medical physics. STAR is not yet well standardized and varies with the experience of the center. The aim of this review is to summarize the main radiobiological and clinical evidence of STAR and discuss emerging applications.

## 2 Radiobiological mechanisms

Since the first report describing the use of STAR for the treatment of refractory VT (10), considerable progress has been made in elucidating the biological mechanisms underlying its therapeutic effects. Initially, STAR was conceived as a non-invasive alternative to catheter ablation, based on the assumption that high-dose irradiation would reproduce the substrate-modifying effects of thermal ablation through radiation-induced myocardial injury and subsequent fibrosis (11–14). Drawing on decades of experience in oncology and stereotactic radiosurgery, the prevailing paradigm held that high-dose radiation delivered to the arrhythmogenic substrate would initiate a cascade of vascular, inflammatory, and cellular events culminating in scar formation and permanent interruption of re-entrant circuits. This hypothesis was strongly supported by early preclinical investigations. Animal studies demonstrated that very high radiation doses (40–160 Gy) induced transmural myocardial fibrosis and, in some cases, atrioventricular conduction block, whereas substantially lower doses failed to produce comparable structural changes (15, 16). Together with the extensive clinical experience of stereotactic body radiotherapy (SBRT) and stereotactic radiosurgery (SRS) in oncology (17), these findings provided the biological rationale for selecting a single 25 Gy fraction for the first clinical STAR treatments. However, clinical observations that followed have challenged this fibrosis-centered paradigm. Contrary to expectations, patients frequently experienced a marked reduction in ventricular arrhythmias within days to weeks after treatment (10), a time course incompatible with the delayed development of mature fibrosis. These unexpected findings suggested that myocardial scar formation alone could not fully explain the rapid anti-arrhythmic effects of STAR and prompted the search for alternative biological mechanisms (**Figure 1**).

**Figure 1:**
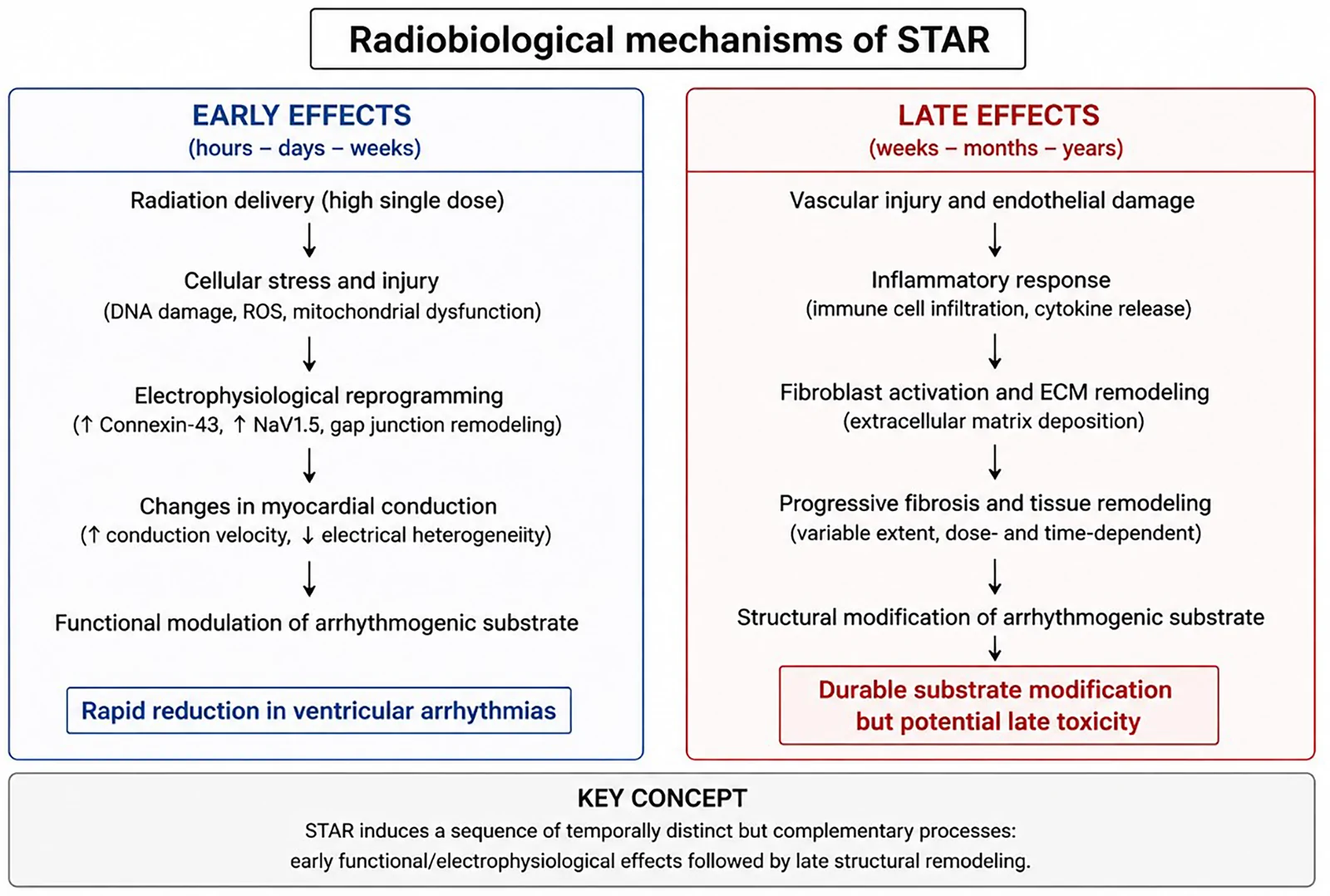
Early and late radiobiological mechanisms of star.

### 2.1 Early and acute effects

Early after irradiation, ionizing radiation induces cellular stress and injury through direct DNA damage and the generation of reactive oxygen species, with potential effects on cardiomyocyte structure, mitochondrial function, and the myocardial microvasculature (11, 15, 16). Experimental studies have also demonstrated early alterations in cardiomyocyte proteins involved in electrical conduction. Zhang et al. (18), using murine models and explanted human cardiac tissue from patients previously treated with STAR, demonstrated persistent upregulation of key cardiac conduction proteins, including connexin-43 (Cx43) and NaV1.5, accompanied by accelerated ventricular conduction. Cx43 is the principal gap-junction protein in ventricular cardiomyocytes, whereas NaV1.5 is the predominant voltage-gated sodium channel responsible for rapid cardiomyocyte depolarization. These findings support the concept that high-dose irradiation can induce electrophysiological reprogramming of the myocardium, modifying conduction properties independently of structural scar formation. By increasing conduction velocity and reducing electrical heterogeneity within the irradiated substrate, these radiation-induced changes may interfere with the conditions required for sustained re-entry and thereby contribute to the rapid anti-arrhythmic effects observed clinically. Importantly, the same experimental model suggested that this response is dose-dependent: single fractions of 15 and 20 Gy produced similar electrophysiological changes, whereas doses of 10 Gy or lower failed to produce comparable effects (19). Consistent with these observations, other preclinical studies have reported early increases in Cx43 expression and gap-junction formation following cardiac irradiation (19). Collectively, these findings suggest that the early response to STAR involves functional remodeling of cardiomyocyte electrical properties that may precede substantial structural remodeling.

### 2.2 Late and chronic effects

The early electrophysiological response is followed by a more prolonged process of tissue remodeling involving vascular injury, inflammatory responses, extracellular-matrix remodeling, fibroblast activation, and fibrosis (20). The magnitude and distribution of these structural changes appear to depend on radiation dose, time from irradiation, and the experimental model. Very high radiation doses, particularly those exceeding approximately 35–40 Gy, can produce extensive myocardial fibrosis and, in some models, injury to the cardiac conduction system (21). Preclinical studies summarized by Galietta et al. (19) have described a spectrum of histopathological findings following STAR, including myocyte architectural disruption, vacuolization, inflammatory changes, fibrosis, and extracellular-matrix remodeling. Importantly, the development of fibrosis following clinically relevant doses is neither immediate nor uniform. Whitaker et al. (20) emphasized that late fibrosis may become apparent approximately 3–4 months after cardiac irradiation and may not fully account for either the early or late anti-arrhythmic effects of STAR. Thus, late substrate modification may result from a combination of progressive fibrosis, altered myocardial architecture, and persistent changes in cellular and intercellular electrophysiological properties rather than from scar formation alone. In support of progressive structural remodeling, Benali et al. (22) reported a reduction in bipolar voltage within myocardial regions receiving at least 15 Gy in an *in vivo* electroanatomical mapping study performed eight months after STAR. Although limited to a single patient, these findings suggest that delayed structural remodeling may progressively develop after the initial electrophysiological response. Taken together, the available evidence supports a model in which STAR induces a sequence of temporally distinct but potentially complementary biological processes. The early phase, occurring within days to weeks after irradiation, appears to involve radiation-induced cellular stress and electrophysiological reprogramming, including changes in Cx43, NaV1.5, gap-junction organization, and myocardial conduction. These changes may contribute to the rapid suppression of ventricular arrhythmias. The subsequent late phase is characterized by vascular and inflammatory responses, extracellular-matrix remodeling, fibroblast activation, and variable degrees of fibrosis, which may contribute to more durable substrate modification but may also underlie late radiation-induced cardiac toxicity (20).

### 2.3 Implications for treatment optimization

This evolving biological model has important implications for treatment optimization. Rather than defining STAR simply as a non-invasive form of catheter ablation, it may be more appropriate to consider it a therapy that combines functional electrical modulation with delayed structural remodeling. Understanding the relative contribution of these mechanisms is essential for identifying the minimum radiation dose capable of achieving durable anti-arrhythmic efficacy while minimizing long-term toxicity. Several important biological questions nevertheless remain unanswered. To date, no studies have specifically investigated the contribution of immune-mediated mechanisms following cardiac irradiation, despite the inflammatory cascade that is inevitably triggered after radiation exposure (23). Likewise, the radiobiology of the irradiated myocardium remains incompletely characterized. In particular, the α/β ratio of cardiac tissue following single-fraction irradiation is unknown, limiting the ability to define a truly ablative dose for dysfunctional myocardium and to accurately estimate radiation tolerance of individual cardiac substructures using the linear-quadratic (LQ) model (24). Although an α/β value of approximately 3 Gy is commonly assumed for late-responding tissues, experimental studies in rat hearts have suggested considerably lower values, potentially approaching 1 Gy (25, 26).

These uncertainties have direct clinical implications. If electrophysiological reprogramming is primarily responsible for the therapeutic effects of STAR, whereas vascular injury and fibrosis contribute predominantly to late toxicity, the optimal radiation dose may not necessarily coincide with the dose traditionally considered ablative. This hypothesis raises the possibility that lower single-fraction doses or hypofractionated schedules could preserve anti-arrhythmic efficacy while reducing long-term cardiac injury. Unlike conventional catheter ablation, radiation therapy offers considerable flexibility in dose and fractionation, and there is no universal ablative regimen that simultaneously maximizes therapeutic efficacy while minimizing toxicity to surrounding cardiac and extracardiac tissues. Indeed, in radiation oncology, hypofractionated regimens delivered over three fractions are frequently adopted because they provide a favorable balance between efficacy and normal tissue tolerance (27). Whether similar strategies could improve the therapeutic index of STAR remains an important unanswered question.

### 2.4 Potential role of vascular injury

To date, the contribution of vascular injury to both the therapeutic efficacy and long-term toxicity of STAR has not been directly investigated. Nevertheless, extensive evidence from radiation oncology demonstrates that radiation exposure is an independent risk factor for cardiovascular morbidity and mortality following conventionally fractionated thoracic radiotherapy (28). Radiation-induced endothelial dysfunction, inflammation, thrombogenicity, and microvascular injury may lead to tissue ischemia, capillary rarefaction, and fibrosis (29–32). Evidence from SRS further supports the radiosensitivity of the vascular endothelium. In this setting, radiation primarily induces endothelial cell apoptosis, microvascular dysfunction, secondary inflammatory responses, including T-cell infiltration, and vascular occlusion (33, 34). Several studies have demonstrated a clear dose-dependent vascular response (35). Single-fraction doses above 10 Gy are generally considered capable of inducing substantial vascular injury (36). These observations are particularly relevant to STAR, which typically delivers high-dose radiation in a single fraction to the myocardium. A recent preclinical study demonstrated that delivery of a single 16 Gy fraction to different cardiac regions in mice produced both structural and functional abnormalities, with the greatest and most persistent alterations observed after irradiation of the cardiac base, suggesting regional differences in cardiac radiosensitivity (37). Likewise, clinical studies in patients with non-small cell lung cancer treated with SBRT have reported clinically significant cardiac dysfunction within two years after treatment, which was associated with increased non-cancer mortality (38). Although these findings cannot be directly extrapolated to STAR, they suggest that exposure of cardiac tissues to high single-fraction radiation doses has the potential to induce persistent vascular injury, and this fact is particularly relevant in patients with refractory VT who frequently have underlying ischemic cardiomyopathy or diffuse coronary artery disease already compromising the cardiac vascular reserve. In these patients, additional radiation-induced microvascular dysfunction could theoretically exacerbate myocardial ischemia, accelerate adverse ventricular remodeling, and ultimately compromise long-term cardiac function and survival. These uncertainties raise the necessity to explore strategies aimed at minimizing vascular injury while preserving anti-arrhythmic efficacy and improving the therapeutic index of STAR. Both dose de-escalation and hypofractionated treatment schedules deserve systematic investigation. From a radiobiological perspective, fractionation is well established to reduce late toxicity in normal tissues, including the vasculature, by allowing repair of sublethal damage between fractions while maintaining biological effectiveness (39). Whether these radiobiological principles can be successfully translated to cardiac radioablation, preserving efficacy while reducing late vascular toxicity, represents an important unanswered question that warrants prospective preclinical and clinical investigation. Assessment at baseline and at prespecified longitudinal intervals after STAR of circulating biomarkers of myocardial injury, inflammation, endothelial dysfunction, and fibrosis may provide complementary measures of subclinical toxicity, help distinguish acute from delayed effects and contribute to establishing dose–response relationships between vascular injury, cardiac dysfunction, and arrhythmic outcomes. Additionally, follow-up in these patients should include serial evaluation of left and right ventricular function, myocardial perfusion, and structural changes using echocardiography and cardiac MRI, with particular attention to regional changes within or adjacent to the irradiated volume.

Overall, the available evidence suggests that the early anti-arrhythmic effects of STAR are more likely to be driven by radiation-induced electrophysiological reprogramming and conduction remodeling than by vascular injury. In contrast, vascular damage may play a critical role in determining the long-term safety profile of the treatment. Defining the dose–response relationship between electrophysiological efficacy and vascular toxicity should therefore represent a major priority for future translational and clinical research (**Figure 2**).

**Figure 2:**
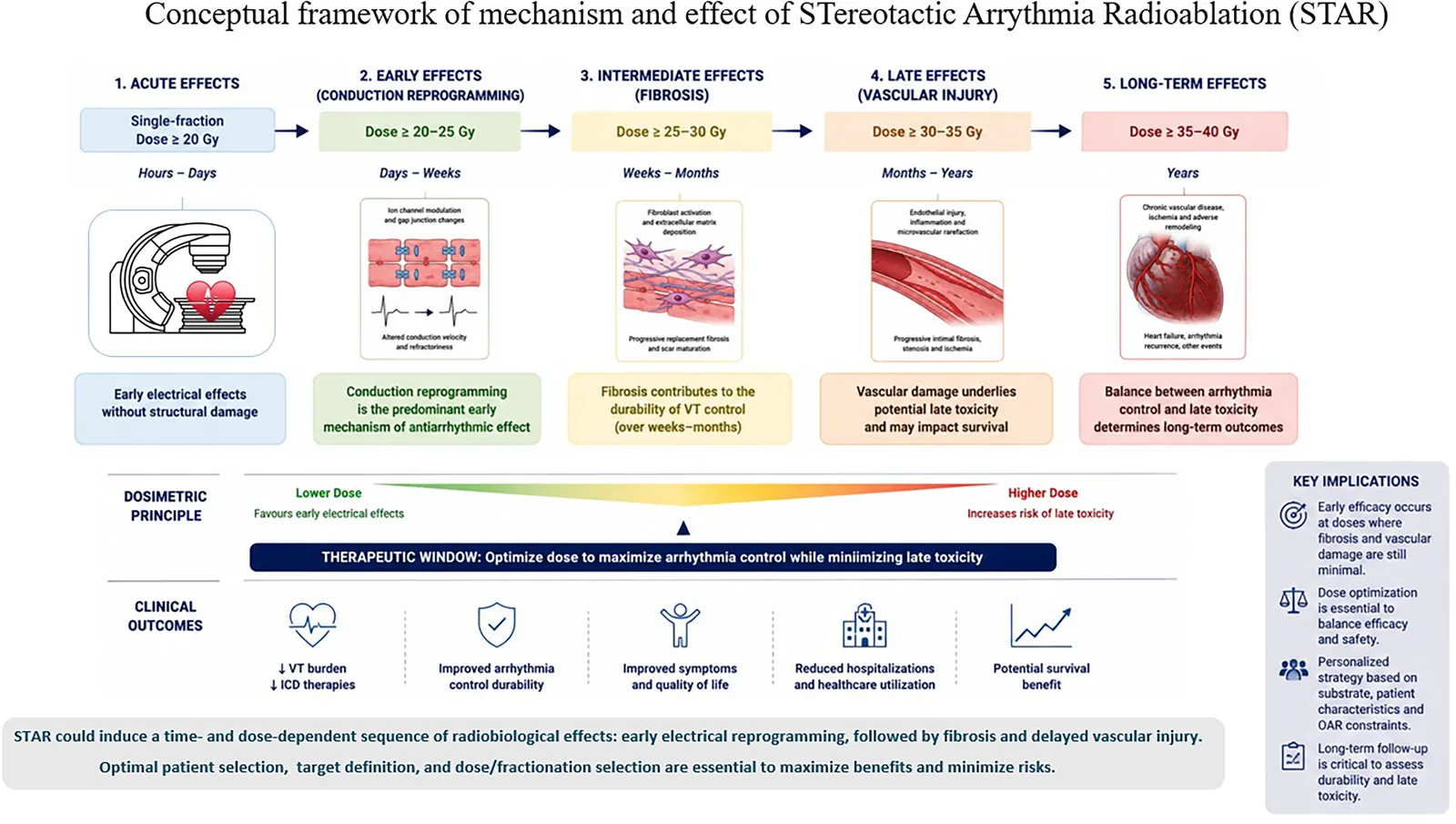
Conceptual framework of mechanism and effect of stereotactic arrhythmia radioablation (STAR).

## 3 Clinical evidence

### 3.1 Efficacy and safety

Currently, prospective clinical evidence supporting STAR remains limited, with relatively small sample sizes and short or heterogeneous follow-up durations across individual studies. These limitations highlight the need for international collaborative efforts to generate larger, harmonized datasets and provide more robust evidence regarding the efficacy and safety of STAR. The prospective, multicenter STOPSTORM.eu registry (9) was established to address this need and included 193 patients with refractory VT treated with STAR (median radiation dose was 25 Gy delivered in a single fraction) across 28 European centers in its interim analysis (9). Among the 107 patients with at least 6 months of follow-up, the median reduction in VT burden during the 6 months after STAR compared with the 6 months before treatment was 80%. However, VT recurrence occurred in 55% of the overall cohort during follow-up. A clinically relevant reduction in ICD shocks was also observed, with 72% of evaluable patients remaining free from ICD shocks during the first 6 months after STAR. At 6 and 12 months post-STAR, 83% and 64% of the complete cohort were still under follow-up, with survival probabilities of 88% and 77%, respectively, reflecting the advanced underlying cardiac disease in this population. Regarding safety, 12 serious adverse events were evaluated as possibly or probably related to treatment, including pericardial effusion, coronary events, and early post-treatment ventricular arrhythmia. No deaths were considered treatment-related. Overall, the STOPSTORM registry provides the largest multicenter evidence to date supporting STAR as a potentially effective treatment for reducing VT burden and ICD shocks in patients with refractory VT. Nevertheless, the persistence of arrhythmic recurrences, the high mortality of the underlying population, and the absence of a randomized control group highlight the need for further controlled studies to better define the efficacy, safety, and optimal clinical role of STAR.

Among the prospective studies, Robinson and colleagues’ single-arm phase I/II trial, ENCORE-VT (40), represents one of the earliest and largest clinical trials (19 patients) evaluating STAR reporting results at the longest follow up (median time of 24 months) (41). ENCORE-VT evaluated the efficacy of STAR guided by a non-invasive electrophysiological mapping approach in a cohort of 19 patients with refractory VT. The study reported a significant reduction in the number of aggregate VT episodes comparing the 6 months before treatment and the 6 weeks and 6 months after treatment and a significant reduction of the median number of VT episodes comparing 6 months pre- and post-STAR. The response rate in terms of aggregate VT episode reduction was 94% (1778 vs 111) comparing 6 months pre- and post-procedure, with 149 VT episodes recorded 6 weeks after SBRT. The median number of VT episodes decreased from 199 episodes (range 4-292) in the 6 months pre-ablation period, to 3 episodes (range 0-31) during 6-month post-ablation period.). In the initial report, overall survival was 89% and 72% at 6 and 12 months, respectively. Six deaths were reported, including two considered possibly related to treatment, occurring at 8.3 and 15 months after STAR (40). During the first 90 days following treatment, two grade 3 treatment-related serious adverse events were reported: one case of heart failure exacerbation at 65 days and one case of pericarditis requiring hospitalization at 80 days. At a median follow-up of 24.1 months (range, 12.8–28.8 months), the same study group subsequently reported an overall survival of 58%, with eight deaths, four of which were considered possibly treatment-related (2 heart failure, 2 VT recurrence) (41). Late (>6 months) serious adverse events considered probably or definitely treatment-related included a grade 3 pericardial effusion occurring at 2.2 years and a grade 4 gastro-pericardial fistula occurring at 2.4 years and requiring surgical repair. More recently, a systematic review and meta-analysis conducted on behalf of the STOPSTORM.eu consortium (42) identified 10 prospective trials including 82 patients treated with STAR between 2016 and 2022, showing substantial reductions in VT burden (≥50% reduction in 90% of evaluable patients) with moderate toxicity (10% grade ≥3 adverse events at 90 days). However, 1-year outcomes remained limited, with a recurrence-free survival (RFS) of 21% and an OS of 73%. Another debated point remains the definition of optimal duration of a post-STAR blanking period. While the majority of STAR studies adopted a 6-week blanking period, the current EHRA/HRS consensus recommends reporting all ventricular arrhythmic events without applying a blanking period (43). In the STOPSTORM registry, 52% of all VT episodes occurring within the first 6 months were recorded during the first 6 weeks after STAR, with ICD shocks also clustering during this early period, supporting the concept of an initial blanking phase. Nevertheless, the delayed and potentially heterogeneous radiobiological effects of radiation make the optimal duration of such a window difficult to define, and further studies are needed to establish a standardized approach. The findings from the most relevant prospective studies (9, 40, 41, 44–53), including at least 5 treated subjects, are summarized in **Table 1**.

**Table 1:** Findings from the most relevant prospective studies.

Study [publication year]	No. of enrolled patients	Age (years)	Etiology (ICM/NICM)	No. of pre CA	NYHA class- %				Radiation technology	Dose (Gy)	PTV (CTV) (ml)	Blanking window	VT burden reduction	Follow up	Overall deaths in a year	Complications related to STAR
					I	II	III	IV								
Robinson et al (ENCORE-VT) [2019] (40, 41)	19	66 (61-78)	11/8	0-4	5.3	21.1	52.6	21.1	Linear accelerator	25	60.9–298.8 (6.4–88.6)	6 weeks	94%	Median: 6 months	6	1 pericarditis; 1 heart failure (possible)
														Median: 24.1 months	8	1 grade 3 pericardial effusion; 1 grade 4 gastro-pericardial fistula 4 grade 5 (2 heart failure, 2 VT recurrence)
Gianni et al. [2020] (44)	5	62	4/1	1-2	20	80	0	0	Cyberknife	25	80–184	n/a	No reduction	Median:12 ± 2 months	2	None
Carbucicchio et al. (STRA-MI-VT) [2021] (45)	7	70 ± 7	4/3	0-3	29	71	0	0	Linear accelerator	25	88.1–239 (16.04–55)	6 weeks	93%	4 pt complete 6 months FU	3	1 nausea/vomiting 1 pulmonary fibrosis
Lee et al. [2021] (46)	7	60s–70s	5/2	0-3	0	43	43	14	Linear accelerator	25	57.5–139 (n/a)	n/a	85%	Median: 6 months	1	None
Qian et al. [2022] (47)	6	72 (70–73)	6/0	2-4	–	–	–	–	Linear accelerator	25	170.5–443.9 (70.5–247.3)	None	31%	Median: 231 days	3	1 pneumonitis 1 heart failure 1 moderate pericardial effusion
van der Ree et al. (STARNL-1) [2023] (48)	6	54-83	6/0	1-5	0	50	50	0	Linear accelerator	25	93–372 (15–87)	6 weeks	>50%	Range: 7–12 months	2	1 grade 3 intracardiac thrombus + 26 adverse events grade 1 and grade 2
Miszczyk et al. (SMART-VT) [2023] (49)	11	45-72	9/2	1-4	10	72	18	0	Linear accelerator	25	18.6–111.3 (n/a)	3 months	84.3%	Range: 3.5–20.3	2	1 grade 4 heart failure
Krug et al. (RAVENTA) [2023] (50)	5	49-74	2/3	0-6	–	–	–	–	Linear accelerator	25	43.4–80.7 (6–34.4)	6 weeks	3 pts VT reduction; 1 pt VT recurrence; 1 died	Median: 6 months	1	None
Chang et al. [2023] (51)	6	63-85	3/3	0-2	0	0	33	67	Linear accelerator	25	17.5–246.8 (3–78.6)	None	3 pts complete remission; 2 pts partial remission; 1 pt VT recurrence	Median: 12.3 months	1	None
Arkles et al. [2024] (52)	14	65 ± 7.8	–	2 ± 1.1	–	–	–	–	Linear accelerator	25	84.9 ± 24.4 (45.6 ± 13.0)	6 weeks	98%	Range: 6–12 months	4	None
Kabuki et al. [2026] (53)	8	69 (49-91)	2/6	5	0	37.5	37.5	25	Linear Accelerator/Carbon-ion	25	49.7–117.1 (8.3–37.9)	n/a	97.9%	Median: 2.2 years	2	1 nausea
STOPSTORM registry [2026] (9)	193	68 ± 9	90/103	1-3	16	45	32	8	Linear accelerator (including MR Linac); Robotic Linac	25	61-141 (n/a)	6 weeks	80%	Median: 19 months	43	12 serious adverse events possibly or probably treatment-related (no grade 5)

Despite the growing number of ongoing and recently reported clinical trials, robust large-scale datasets remain lacking. Further prospective studies with longer follow-up are required to better define the efficacy, characterize late toxicities, optimize safety, and promote standardization of STAR treatment approaches (54, 55).

### 3.2 Target definition

An important variable that has not been well-established in STAR protocols is the procedure for target delineation (56). In this field, the target for radioablation should encompass the arrhythmogenic substrate within the heart. Considering that it is defined primarily by electrical properties, there is no directly visible “gross” disease for contouring that could help to reduce interobserver variability. However, abnormal VT isthmuses are frequently located within or adjacent to regions of myocardial fibrosis, and identification of scar can therefore provide an important anatomic surrogate for target delineation. Moreover, defining a common language for targeting is critical, particularly when bringing together disparate specialties such as radiation oncology and electrophysiology, which often think in different anatomic views and use different nomenclature (56). Invasive 3D electroanatomic mapping (EAM) provides an accurate means of identifying arrhythmogenic substrate and characterizing circuits to be targeted with catheter ablation. The use of electrocardiographic imaging (ECGI) in combination with standard cardiac imaging, such as cardiac magnetic resonance (CMR) and CT with contrast, has been used as a noninvasive alternative to EAM for VT source localization although its spatial resolution is lower than that of EAM (57). Multimodality substrate characterization is increasingly being incorporated into STAR target definition. CMR, particularly late gadolinium enhancement (LGE), provides detailed tissue characterization and allows identification of myocardial fibrosis, including scar core and border-zone regions that may harbor potential conducting channels and components of the arrhythmogenic substrate. Integration of CMR-derived scar information with 3D EAM, CT, and ECG data can therefore provide complementary anatomical and electrophysiological information for individualized target delineation (58).

More recently, advanced image registration and segmentation platforms have facilitated this multimodality approach by generating patient-specific three-dimensional (3D) models of cardiac anatomy and myocardial substrate and integrating imaging-derived information with electroanatomic data. These platforms allow electroanatomic mapping data to be incorporated into radiation treatment planning systems, providing an individualized framework for clinical target volume (CTV) delineation and treatment planning (59). In particular, dedicated segmentation platforms such as MUSIC/inHEART enable the integration of multimodality cardiac imaging and electrophysiological data to generate 3D representations of the arrhythmogenic substrate and surrounding cardiac structures, potentially improving the consistency and reproducibility of target definition (60). Importantly, accurate transfer of the arrhythmogenic target from EAM and cardiac imaging to the treatment-planning CT remains a critical step in STAR planning. Recent software developments include semi-automated segmentation based on the American Heart Association 17-segment model, as well as 2D-to-3D and 3D-to-3D target-transfer approaches designed to improve the reproducibility of target localization (61). However, differences in image acquisition, spatial resolution, cardiac and respiratory motion, and registration methods can introduce uncertainty during multimodality co-registration, and a standardized workflow for CTV definition and target transfer has not yet been established (62). In this context, accurate identification of the arrhythmogenic substrate and its translation onto the simulation CT used for SBRT treatment planning require close collaboration between electrophysiology and radiation oncology. Standardization of imaging acquisition, image registration, target definition, and reporting will be essential to reduce interobserver variability and facilitate reproducible STAR treatment planning.

### 3.3 Weill Cornell Medicine experience and prospective trial

At Weill Cornell Medicine (WCM), between September 2019 and April 2023, five patients with refractory ventricular tachycardia (VT) underwent STAR treatment. The clinical target volume (CTV) was defined by a multidisciplinary team of radiation oncologists and cardiac electrophysiologists based on EAM data, cardiac CT imaging, and prior catheter ablation maps. To improve target delineation, we implemented the use of a dedicated software platform (inHEART, inHEART Medical, Pessac, France), which integrates three-dimensional EAM data with contrast-enhanced cardiac CT to identify the arrhythmogenic substrate and generate a treatment target suitable for SBRT planning (**Figure 3**). The resulting cardiac CT dataset was subsequently co-registered with the simulation CT, allowing accurate transfer of the target volume to the treatment planning system. An internal target volume (ITV) was generated to account for cardiac and respiratory motion, and the planning target volume (PTV) was created by applying a 2–3 mm isotropic expansion to the ITV. Volumetric Modulated Arc Therapy (VMAT) plans were generated, optimized, and delivered using a TrueBeam™ (Varian Medical System) linear accelerator employing both cone beam CT and surface-guided radiotherapy for real-time image guidance. The prescription dose to the PTV was 25 Gy in 1 fraction. Mean CTV, ITV and PTV volumes, were 141.1cc, 187.7cc, 298.1cc, respectively. Mean heart volume was 1740.6 cc. The main dosimetric data are summarized in **Table 2**. There were no acute treatment-related adverse events. Clinical and treatment-efficacy data are summarized in **Table 3**. STAR significantly reduced or abrogated arrhythmia at a median time of 24 weeks (range 4-48) post-treatment. Patient n.1 and n.3 showed a remarkable reduction of VT episodes at 4- and 8-week post-treatment, respectively. Patients n.2, n.4 and n.5 were free of VT episodes at 6-month post-treatment. At a mean follow-up of 18 months (range: 1–50 months), no late adverse events were recorded. One of five patients (Patient 2) remained alive at 50 months post-STAR, free of VT events at 4 years post-treatment. Patient n.1 died due to complication after cardiac transplantation, patients n.3 due to sepsis and multiorgan failure and patient n.5 due to COVID pneumonia, at 11-, 1- and 6-months post-STAR, respectively. Patient 4 remained free of VT events at 16 months post-treatment but subsequently developed a VT storm with multiple implantable ICD shocks and underwent repeat VT catheter ablation. At 21 months post-STAR, the patient was admitted with cardiogenic shock in the setting of advanced heart failure and died during hospitalization despite intensive cardio-vascular supportive cares. This retrospective series of five high-risk patients with VT refractory to catheter ablation and medical therapy, treated with STAR at a single referral center, suggests that LINAC-based STAR is a safe and effective treatment option in this population. These findings supported the development of a phase I/II randomized trial at the same institution.

**Figure 3:**
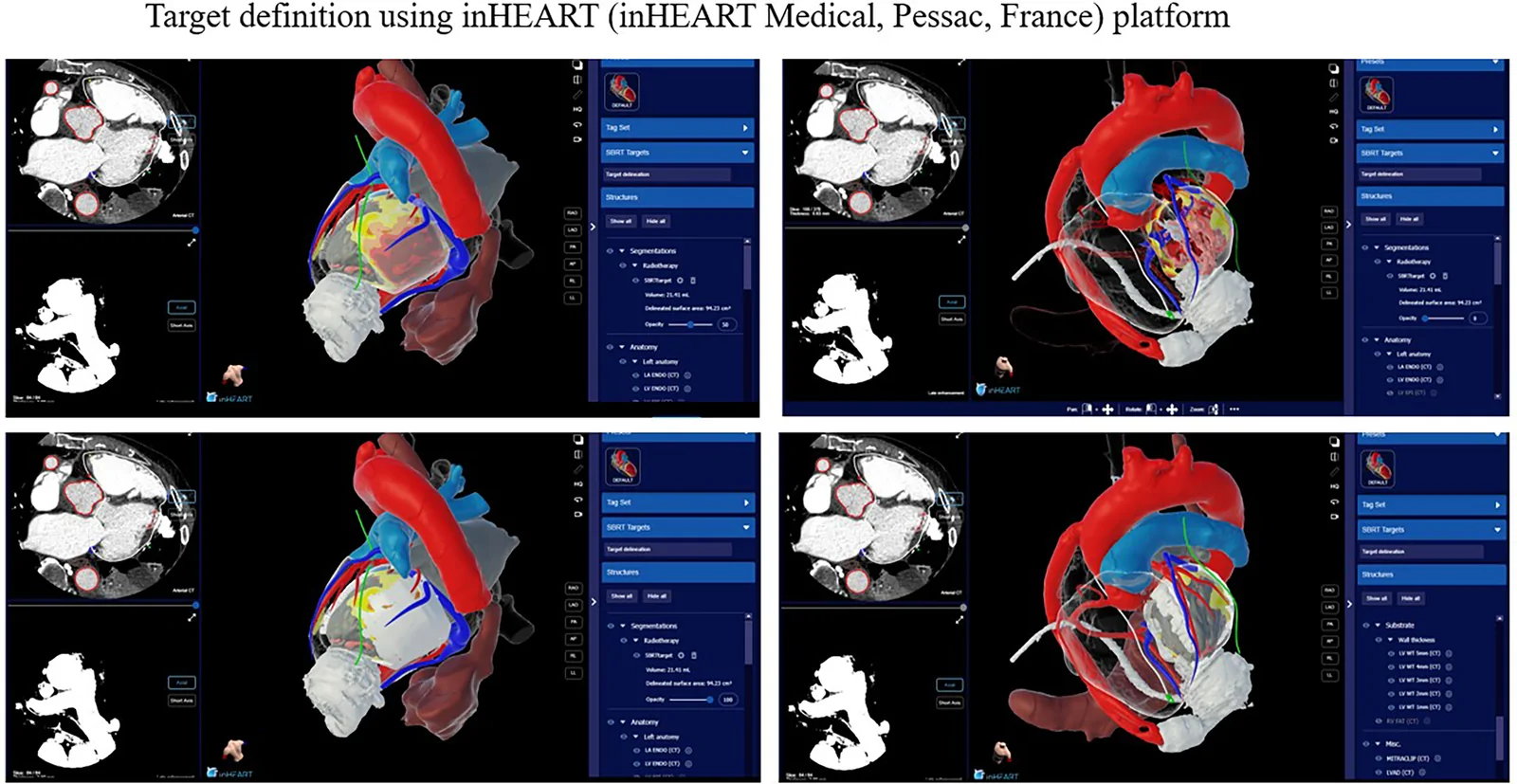
Target definition using inHEART (inHEART Medical, Pessac, France) platform.

**Table 2:** Dosimetric data summary.

Patient	CTV (cc)	ITV (cc)	PTV (cc)	Heart volume (cc)	PTV Dmax (Gy)	Heart Dmean (Gy)	LAD Dmean (Gy)	Esophagus Dmax (Gy)	Stomach Dmax (Gy)	Main Bronchus (Gy)
#1	130.3	na	178.2	2664.5	28.48	6.42	na	13.53	na	9.40
#2	30.2	91	205.3	1512.9	31.25	10.64	18.74	14.67	0.39	16.23
#3	378.6	378.6	609.2	1983.1	33.39	13.40	5.27	12.90	19.80	7.45
#4	143.2	200.4	345.3	1154.2	32.49	13.47	8.13	8.47	21.73	15.93
#5	23.1	81	152.7	1388.3	34.21	10.48	4.22	13.53	1.28	6.26

**Table 3:** Clinical and treatment efficacy data summary.

Patient	Treatment	Follow up	Status	Arrhythmia condition pre-STAR	Arrhythmia condition post-STAR	Cause of death
#1	09/2019	11 months	Deceased	16 treated VT/VF episodes	2 VT episodes within 2 months post treatment	Complication after cardiac transplantation
#2	05/2022	50 months	Alive	5 ICD shocks and 3 catheter ablations	0 VT episodes at 6, 12, 24, 36, and 48-months post-treatment	–
#3	09/2022	1 month	Deceased	>30 VT therapies and VT storm	Remarkable reduction of VT episodes at 4 weeks post treatment	Sepsis, multiorgan failure
#4	01/2023	21 months	Deceased	>30 ICD shocks and VT storm	0 VT episodes at 6,12 and 16-months post-treatment	Cardiogenic shock in advanced heart failure
#5	04/2023	6 months	Deceased	27 VT therapies and 107 VT episodes	0 VT episodes at 6 months post treatment	COVID pneumonia

Building on our institutional experience, the available clinical evidence, and the remaining knowledge gaps in the field, we have designed a prospective randomized phase I/II trial to address one of the major unresolved questions in STAR: the optimal radiation dose and fractionation schedule (NCT06743789). The study investigates two complementary dose de-escalation strategies in patients with refractory VT: progressive reduction of the single-fraction dose (20, 15, and 10 Gy) and biologically equivalent three-fraction regimens (36, 30, and 24 Gy). Using a randomized dose de-escalation design, the trial aims to identify the lowest effective and safest regimen based on co-primary efficacy and safety endpoints (**Figure 4**). Beyond evaluating arrhythmia control, the study will provide important prospective data on acute and long-term toxicity, including radiation-induced cardiac and vascular effects. By directly comparing single-fraction dose reduction with hypofractionated treatment, this trial represents the first prospective effort to optimize the therapeutic ratio of STAR and define a safer, evidence-based dose and fractionation strategy for the treatment of refractory VT. This trial is currently open to accrual at WCM.

**Figure 4:**
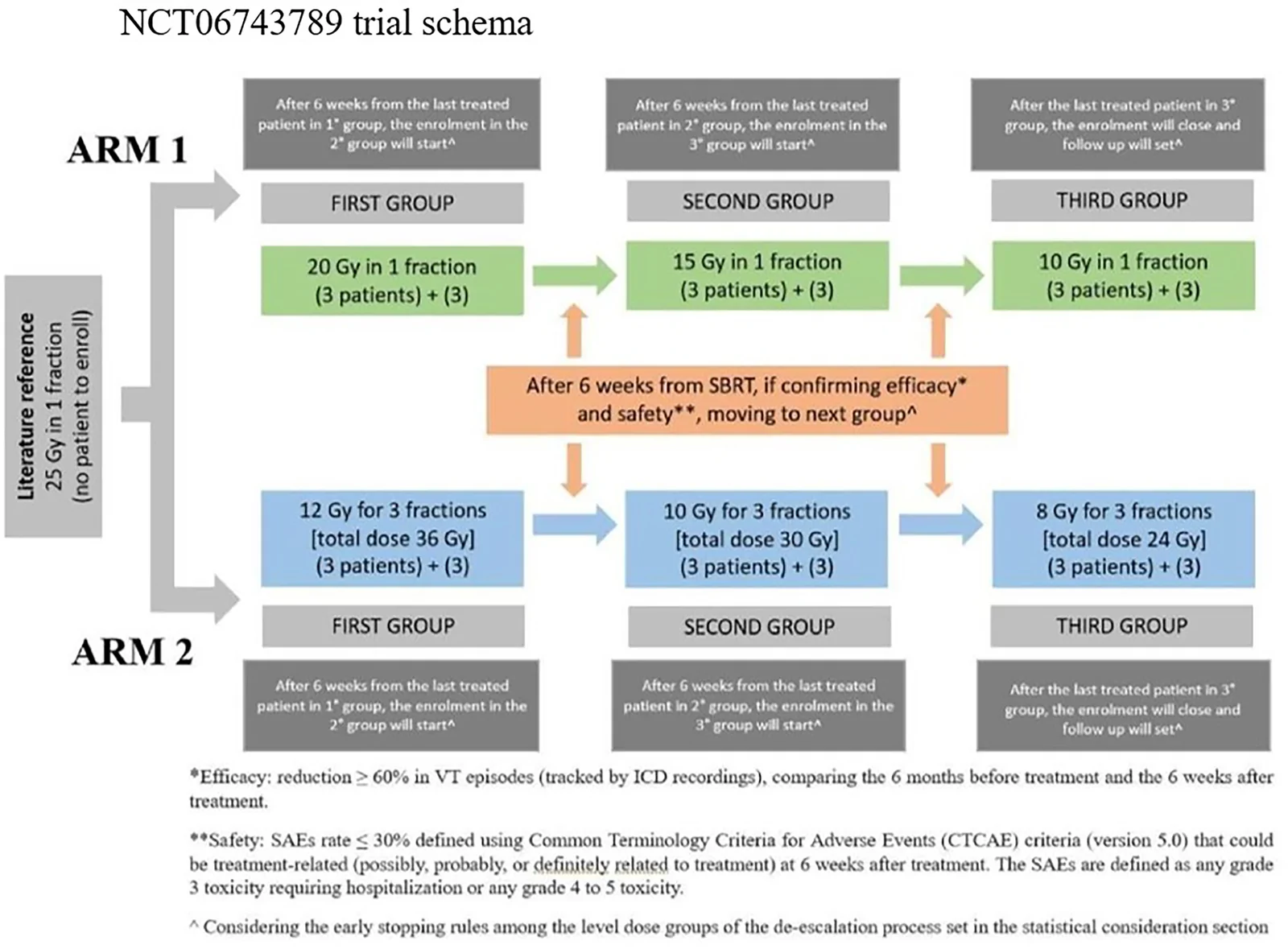
NCT06743789 trial schema *Efficacy: reduction ≥ 60% in VT episodes (tracked by ICD recordings), comparing the 6 months before treatment and the 6 weeks after treatment. **Safety: SAEs rate ≤ 30% defined using Common Terminology Criteria for Adverse Events (CTCAE) criteria (version 5.0) that could be treatment-related (possibly, probably, or definitely related to treatment) at 6 weeks after treatment. The SAEs are defined as any grade 3 toxicity requiring hospitalization or any grade 4 to 5 toxicity. ^ Considering the early stopping rules among the level dose groups of the de-escalation process set in the statistical consideration section.

## 4 Emerging applications

### 4.1 Future directions

The available evidence supports STAR as a promising therapeutic option for patients with refractory VT. However, patient selection remains an important area of uncertainty, and currently available evidence does not support a definitive algorithm for identifying patients who are most likely to benefit from STAR. Nevertheless, several clinical features may help identify patients in whom STAR should be considered, particularly those with recurrent VT despite prior catheter ablation, multiple ICD therapies, or electrical storm despite optimized medical and interventional management. The outcome and feasibility of previous ablation attempts should also be considered, including failure to adequately modify the arrhythmogenic substrate, VT recurrence despite apparently successful ablation, or substrates that are difficult or unsafe to access using conventional endocardial or epicardial approaches. In these selected patients, particularly when the risks of repeat invasive procedures are substantial or conventional treatment options are limited, STAR may represent a valuable alternative or adjunctive treatment strategy (43). Further prospective studies are needed to define clinical, electrophysiological, imaging, and procedural characteristics that best predict benefit from STAR and to clarify its optional position within the treatment pathway relative to repeat catheter ablation. At the same time, the relatively high rates of VT recurrence post STAR reported in some series, limited clinical experience, and paucity of long-term follow-up warrant careful consideration of both the potential benefits and risks of STAR, including late toxicities that may not yet be fully characterized (41). Well-designed randomized controlled trials with extended follow-up are therefore essential to determine the durability of STAR compared with catheter ablation and antiarrhythmic drug therapy, while also defining its long-term toxicity profile, impact on survival, and effects on patient-reported outcomes (54, 55). A deeper understanding of the biological mechanisms underlying STAR, including the relative contributions of acute electrical conduction reprogramming, delayed radiation-induced fibrosis, and myocardial remodeling, will be equally important to optimize treatment efficacy, improve patient selection, and minimize adverse effects. Several prospective clinical trials are currently addressing these unmet needs. The multicenter randomized RADIATE-VT trial (NCT05765175) is comparing STAR using the Varian Cardiac RadioAblation platform with repeat catheter ablation in patients with high-risk recurrent VT eligible for another invasive procedure, while the randomized CARA-VT trial (NCT05047198) is directly evaluating STAR against conventional catheter ablation. These studies are expected to provide the higher levels of evidence regarding the comparative efficacy, safety, and impact of STAR on clinically meaningful outcomes, including VT burden, ICD therapies, quality of life, and survival. Dose optimization also remains a major research priority. The phase II STAR-VT trial (NCT05258422) is investigating whether reducing the prescription dose to 20 Gy can maintain efficacy while improving the safety profile, whereas the ongoing DEFT-STAR phase II study is evaluating a dose-escalation strategy (NCT05594368). In parallel, as described above, our institutional prospective study is investigating both single-fraction and multi-fraction treatment regimens (NCT06743789) to define the optimal balance between arrhythmia control and radiation-induced toxicity. Beyond individual clinical trials, the international STOPSTORM consortium aims to standardize the clinical implementation of STAR through a prospective multicenter validation cohort. By harmonizing imaging, target delineation, treatment planning, dosimetry, and follow-up protocols, the consortium seeks to generate high-quality evidence, optimize patient selection, and improve the safety, reproducibility, and personalization of STAR. An important future direction is the development of a completely non-invasive treatment pathway, in which the arrhythmogenic substrate is identified and localized using non-invasive electrocardiographic imaging (ECGI) and subsequently treated with STAR without intracardiac catheterization (10, 43). Body-surface potential mapping using multi-electrode vest systems, such as the CardioInsight Noninvasive 3D Mapping System (Medtronic Inc.), has already been used for non-invasive VT source localization and was incorporated into the ENCORE-VT treatment strategy (40), demonstrating a reasonable agreement with invasive electroanatomical mapping (57). The systematic integration of ECGI with STAR could therefore enable a fully non-invasive workflow encompassing arrhythmia characterization, target definition, and treatment, which may be particularly advantageous for frail patients or those at high risk for invasive procedures. Although ECGI currently provides lower spatial resolution than invasive three-dimensional electroanatomical mapping, the spatial accuracy required to define a radiotherapy planning target volume (PTV) may differ from that required to guide catheter positioning. Whether an ECGI-defined target can currently provide sufficient accuracy for STAR treatment planning remains uncertain and requires prospective validation against invasive electroanatomical mapping and clinical outcomes. Future studies should therefore determine the optimal spatial accuracy, target margins, and reproducibility of ECGI-based target definition before this approach can be considered a stand-alone strategy for STAR planning. Collectively, these initiatives are expected to define the optimal role of STAR within the therapeutic algorithm for refractory VT and provide the high-quality evidence needed for its broader clinical adoption.

### 4.2 Technological improvements in radiation treatment techniques

STAR can be performed using different high-precision radiotherapy platforms, with photon-based linear accelerators (LINACs) currently representing the most widely used approach. Conventional LINACs allow treatment with either fixed-field intensity-modulated radiotherapy (IMRT) or volumetric modulated arc therapy (VMAT), providing conformal dose distributions while limiting irradiation of adjacent organs at risk (10, 40, 63, 64). Robotic CyberKnife systems represent an alternative photon-based platform and have been used in both experimental and clinical STAR (21, 44, 65). Their ability to deliver radiation from multiple noncoplanar directions, combined with image-guided robotic positioning, can provide highly conformal treatment and facilitate management of respiratory motion; however, target localization based on orthogonal X-ray imaging may rely on implanted radiopaque structures, such as ICD leads, which may not accurately represent myocardial target motion. In a dosimetric comparison of 13 patients treated with 25 Gy in a single fraction, CK plans demonstrated significantly steeper dose gradients than LINAC plans, with a lower gradient index (3.12 ± 0.71 vs. 3.48 ± 0.55; p = 0.031) and gradient measure (1.00 ± 0.29 vs. 1.17 ± 0.29 cm; p < 0.001), while target coverage and most organ-at-risk doses were comparable. However, CK required substantially longer treatment times than LINAC-based delivery (119.2 ± 43.3 vs. 22.4 ± 1.6 min; p = 0.007), and its dosimetric advantage was more evident for smaller or more spherical targets (65). An additional consideration is target localization, as CyberKnife relies on planar X-ray imaging and may use radiopaque structures, including ICD leads, as surrogates for myocardial target position, which can become less reliable with increasing distance between the lead and the arrhythmogenic substrate (66). MRI-guided LINACs represent an emerging option for STAR because they integrate radiation delivery with continuous soft-tissue visualization. Magnetic resonance-guided radiotherapy may offer important advantages over conventional image-guided radiotherapy by enabling on-table MRI, real-time assessment of organ motion, and adaptation of the treatment plan during treatment (67). In the context of STAR, these capabilities may facilitate more accurate characterization and management of cardiac and respiratory motion, potentially improving target localization and enabling online adaptive treatment strategies. Experimental studies have demonstrated the feasibility of real-time cardiorespiratory motion monitoring and compensation during STAR on MR-LINAC platforms (68). However, clinical experience with MR-guided STAR remains limited, and further clinical studies are needed to determine whether these technological advantages translate into improved treatment accuracy and meaningful clinical benefits. Proton therapy represents another investigational strategy that may provide additional normal-tissue sparing. The finite range of protons and Bragg peak can substantially reduce exit dose and thereby decrease irradiation of surrounding cardiac and extracardiac structures. In a dosimetric analysis of 34 patients with VT, proton plans delivering 25 Gy in a single fraction achieved comparable target coverage while significantly reducing mean heart dose compared with photon plans (3.6 ± 1.5 vs. 5.5 ± 2.0 Gy; p < 0.001); mean lung dose was also reduced from 1.2 to 0.6 Gy (p < 0.001), with additional reductions in the dose to individual cardiac chambers and other critical structures (69). These findings suggest that proton therapy may be particularly advantageous for targets located close to critical cardiac or extracardiac structures. Nevertheless, proton-based STAR remains investigational, with important limitations related to range and relative biological effectiveness uncertainties, sensitivity to cardiac and respiratory motion, limited availability, treatment cost, and potential secondary neutron exposure to cardiac implantable electronic devices (70). Overall, treatment platform selection should be individualized according to target size, location and geometry, proximity to critical structures, motion characteristics, and institutional expertise.

### 4.3 STAR for paroxysmal atrial fibrillation treatment

Although the clinical experience with STAR has largely focused on VT, atrial fibrillation (AF) represents another promising indication for non-invasive cardiac radioablation. Pulmonary vein isolation (PVI), the cornerstone of catheter ablation for AF, remains an invasive procedure that can be challenging in patients with significant comorbidities because of procedural and anesthesia-related risks, including vascular injury, cardiac perforation, heart failure, and hemodynamic compromise. However, the advent of pulsed-field ablation has improved the safety profile of catheter-based AF ablation, with a substantially lower risk of collateral injury to structures such as the esophagus and phrenic nerve compared with thermal ablation (71).hemodynamic In this setting, STAR has emerged as a potential non-invasive alternative capable of achieving electrical isolation of the pulmonary veins without the need for intracardiac instrumentation. Initial clinical experience, including the first-in-human report and the subsequent prospective phase II trial, demonstrated the feasibility of LINAC-based STAR delivered as a single 25 Gy fraction to the pulmonary vein antra, with encouraging safety, significant reductions in AF burden, and marked improvements in quality of life in elderly patients with symptomatic paroxysmal AF (72, 73). Importantly, invasive electro-anatomical mapping performed in patients undergoing electrophysiological study for recurrent atrial arrhythmia confirmed durable pulmonary vein isolation up to 12 months after treatment, providing the first direct electrophysiological evidence that STAR can reproduce the principal therapeutic endpoint of catheter ablation (74). Furthermore, serial echocardiographic evaluation suggested that left atrial reservoir strain may represent a useful biomarker for monitoring atrial functional remodeling after STAR, with lower reservoir strain observed during follow-up in patients experiencing AF recurrence (75). Despite these encouraging findings, the available evidence remains limited to small, single-arm studies with relatively short follow-up, and several anatomical and dosimetric considerations remain important for the development of STAR for AF. Unlike non-target organs, the left atrium and pulmonary vein antra constitute the intended treatment volume; however, the thin atrial wall provides limited separation between the target and adjacent tissues. In particular, the right phrenic nerve courses in close proximity to the right pulmonary veins and therefore represents a potential organ at risk during irradiation of the pulmonary vein antra. Additional concerns relate to the proximity of other radiosensitive structures, including the esophagus, bronchi, and coronary arteries. These anatomical constraints underscore the importance of careful target definition, dose optimization, and treatment planning to achieve adequate pulmonary vein coverage while minimizing radiation exposure to adjacent tissues. Consequently, larger prospective studies with extended follow-up are needed to define the long-term safety profile, optimize patient selection and treatment planning, and determine whether STAR can provide durable rhythm control comparable with conventional catheter ablation while maintaining the potential advantages of a completely non-invasive treatment.

## 5 Conclusion

STAR has emerged as one of the most promising non-invasive treatment strategies for cardiac arrhythmias. Growing preclinical and clinical evidence suggests that its therapeutic effects extend beyond radiation-induced fibrosis, involving early electrophysiological remodeling followed by delayed structural changes. A better understanding of these complementary mechanisms will be essential to optimize treatment efficacy while minimizing long-term toxicity. Although current clinical experience has primarily focused on patients with refractory ventricular tachycardia, the encouraging results achieved in paroxysmal atrial fibrillation demonstrate the potential of STAR beyond ventricular arrhythmias and support further exploration of its role in the treatment of selected cardiac rhythm disorders. At the same time, important questions remain regarding the optimal radiation dose, fractionation, target definition, and long-term safety. Several ongoing prospective studies and international collaborative initiatives are expected to provide more robust evidence on the efficacy, safety, and biological effects of STAR. As radiobiological knowledge and clinical experience continue to evolve, STAR has the potential to become an increasingly refined and personalized therapeutic option, expanding the role of non-invasive STAR in modern cardiac electrophysiology.
